# 3D Culture of Bone Marrow-Derived Mesenchymal Stem Cells (BMSCs) Could Improve Bone Regeneration in 3D-Printed Porous Ti6Al4V Scaffolds

**DOI:** 10.1155/2018/2074021

**Published:** 2018-09-05

**Authors:** Lingjia Yu, Yuanhao Wu, Jieying Liu, Bo Li, Bupeng Ma, Yaqian Li, Zhenfei Huang, Yu He, Hai Wang, Zhihong Wu, Guixing Qiu

**Affiliations:** ^1^Department of Orthopaedic Surgery, Peking Union Medical College Hospital, Peking Union Medical College and Chinese Academy of Medical Sciences, Beijing 100730, China; ^2^Central Laboratory, Peking Union Medical College Hospital, Peking Union Medical College and Chinese Academy of Medical Sciences, Beijing 100730, China; ^3^Department of Orthopaedic Surgery, Fourth Clinical Medical College of Peking University, Beijing Jishuitan Hospital, Beijing 100035, China; ^4^Beijing Key Laboratory for Genetic Research of Bone and Joint Disease, Beijing, China

## Abstract

Mandibular bone defect reconstruction is an urgent challenge due to the requirements for daily eating and facial aesthetics. Three-dimensional- (3D-) printed titanium (Ti) scaffolds could provide patient-specific implants for bone defects. Appropriate load-bearing properties are also required during bone reconstruction, which makes them potential candidates for mandibular bone defect reconstruction implants. However, in clinical practice, the insufficient osteogenesis of the scaffolds needs to be further improved. In this study, we first encapsulated bone marrow-derived mesenchymal stem cells (BMSCs) into Matrigel. Subsequently, the BMSC-containing Matrigels were infiltrated into porous Ti6Al4V scaffolds. The Matrigels in the scaffolds provided a 3D culture environment for the BMSCs, which was important for osteoblast differentiation and new bone formation. Our results showed that rats with a full thickness of critical mandibular defects treated with Matrigel-infiltrated Ti6Al4V scaffolds exhibited better new bone formation than rats with local BMSC injection or Matrigel-treated defects. Our data suggest that Matrigel is able to create a more favorable 3D microenvironment for BMSCs, and Matrigel containing infiltrated BMSCs may be a promising method for enhancing the bone formation properties of 3D-printed Ti6Al4V scaffolds. We suggest that this approach provides an opportunity to further improve the efficiency of stem cell therapy for the treatment of mandibular bone defects.

## 1. Introduction

Mandibular bone defects caused by tumor resection, infection, trauma, or surgery are so common that they consume a large amount of medical resources [1]. Although mandibular reconstructive surgery has been performed for more than a century, the regeneration of critically sized bone defects remains challenging in maxillofacial surgery [2, 3]. Autogenous bone grafting derived from the iliac crest and fibula is the most common procedure for reconstructing mandibular bone defects. However, several limitations restrict its clinical use, including the limited sources of donor tissue, additional surgery, lack of an accurate fit with the defect site, and high rate of donor site infection [4]. Allogeneic bone grafting has also been widely used, but the associated disease transmission and immunogenicity cannot be neglected [5].

Regenerative approaches that avoid autogenous and allogeneic grafts by employing tissue-engineering bone substitutes may provide superior alternative strategies for effective bone reconstruction. Tissue engineering is the use of cells and materials in combination to improve or replace biological tissue, which involves the use of a tissue scaffold. It is well known that the ideal mandibular ramus bone reconstruction implants should have a 3D porous structure with desirable porosity for new bone ingrowth, nutrient diffusion, cell proliferation, capillary infiltration, and appropriate mechanical properties [6].

A large number of tissue-engineering biomaterials, such as collagen, hydroxyapatite (HA), and tricalcium phosphates (TCP), have been developed, but their insufficient mechanical properties and fast degradation rate are unable to meet the clinical demand [7]. With the enormous advantages of 3D printing technology in precision medicine, the 3D-printed porous Ti scaffold showed a superior biocompatibility, high strength-to-weight ratio, lower elastic modulus, and good anticorrosion resistance [8]. However, the inherently insufficient osteogenesis of the Ti makes it difficult for the bone cells to grow into the Ti scaffold. Therefore, scaffolds combined with autologous stem cell tissue have attracted much attention [9, 10]. The major concern with graft cell therapy is the survival rate of cells, which might be improved by the proper 3D extracellular environment [11, 12]. The interaction between stem cells and their surrounding environment is critical for cell behavior. 3D culture may represent a more physiological environment than 2D culture for stem cells, while 2D culture has a dramatically reduced differentiation capacity [13].

In this study, we prepared a 3D-printed porous Ti6Al4V scaffold with customized shape and structure and loaded the Matrigel encapsulating BMSCs into the Ti6Al4V scaffold. This device was designed to provide suitable mechanical properties and a 3D stem cell culture environment. The advantages of our 3D-printed scaffold are the accurate outline dimensions and complex internal morphology of the mandibular ramus, which might meet with the patients' individual requirements [14]. Matrigel has been chosen in our study as the extracellular matrix (ECM) for BMSCs. The ECM is a network tissue that provides mechanical support for stem cells and is essential for stem cells *in vivo*, and it can affect stem cell fate by mediating cell attachment and migration. Several hydrogels, including collagen and laminin, have been produced as an ECM for the 3D culture of stem cells, but Matrigel is regarded as one of the most promising materials that simulates ECM functions [11, 15]. Matrigel can rapidly form a 3D structured hydrogel at 37°C and support cell morphogenesis and differentiation as well as proliferation. It has also been used as a suitable scaffold for supporting stem cells in the treatment of cardiac infarction and arterial injury animal models [16, 17]. Several studies have also reported that on-top Matrigel culture could promote BMSCs osteodifferentiation *in vitro* [18, 19]. However, to date, there have been few reports on the proliferation, osteogenic differentiation, and osteogenesis effects of 3D-cultured BMSCs in Matrigel combined with the Ti6Al4V scaffold *in vivo*.

**Figure 1:**
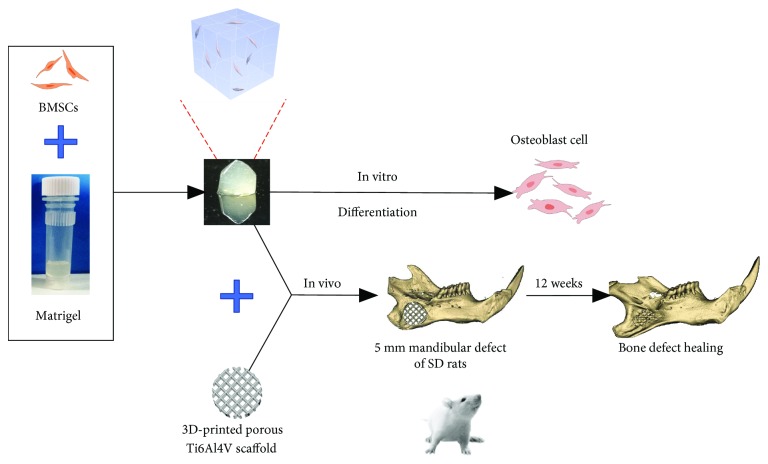
A schematic of the experimental design both *in vivo* and *in vitro*. A schematic of the bone differentiation process of BMSCs encapsulated in the Matrigel *in vitro* and the Ti6Al4V scaffold-Matrigel-BMSC complex device enhancing bone repair *in vivo.*

The goal of this study was to investigate the effects of BMSCs 3D cultured in Matrigel *in vitro* via a cell transplantation viability assay and osteodifferentiation study. Moreover, the efficacy of the combinational strategy of BMSCs, Matrigel, and 3D-printed Ti6Al4V scaffold was evaluated in a full-thickness critical mandibular defect animal model. The experimental process is schematically depicted in Figure 1.

## 2. Methods

### 2.1. 3D-Printed Ti6Al4V Scaffolds

The Ti6Al4V scaffolds were produced as previously reported [5] with minor modifications. Briefly, the scaffolds were produced with Ti6Al4V powder (ASTM B348, grade 23) using selective laser melting (SLM; Concept Laser, Lichtenfels, Germany). The scaffolds were disk shaped with a 5 mm diameter, 1 mm height, 200 *μ*m strut width, and 600 *μ*m pore size. The porosity was 87.4% for animal experiments. All samples underwent a postproduction heat treatment and were verified by micro-CT (SHIMADZU, inspeXio SMX-90CT Plus, Japan).

### 2.2. 2D Cell Culture

**Figure 2:**
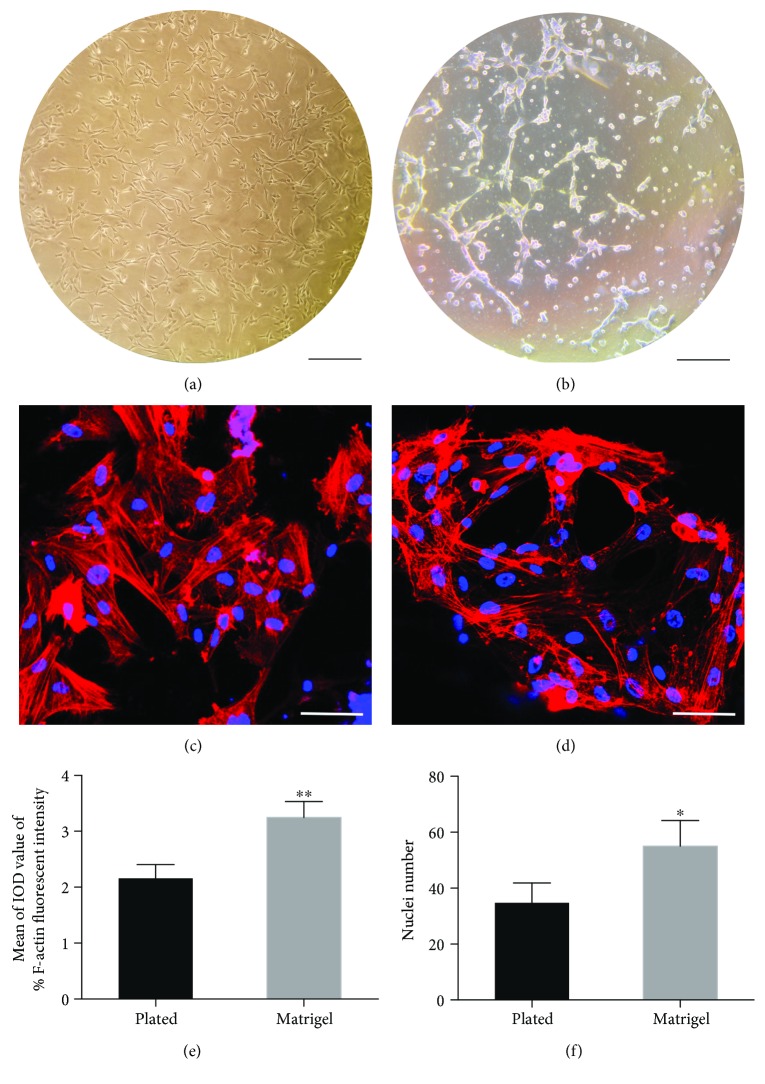
Representation of passage 4 BMSCs on 2D culture plates (a) and in Matrigel 3D culture (b) (scale bar: 300 *μ*m). Representative images of fluorescent staining for F-actin and nuclei (c and d). Blue: DAPI stain, red: rhodamine-phalloidin (scale bar 50 *μ*m). The fluorescent quality of F-actin (e) and the nuclei number (f) was analyzed and compared using ImageJ 1.50i, and the mean of the IOD value was considered as the level of fluorescence intensity. ^∗^ *p* < 0.05, ^∗∗^ *p* < 0.01.

Briefly, human BMSCs (passage 1) were purchased from 307-Ivy Translation Medicine Center (Beijing, China) and grown in hMSC basal media (HUXMA-90011, Cyagen, USA) supplemented with 10% fetal bovine serum (Cyagen, USA) and 1% penicillin-streptomycin (Cyagen, USA) and glutamine (Cyagen, USA) at 37°C with 5% CO_2_ and saturated humidity. We used 0.25% trypsin without EDTA (Solarbio Science & Technology Co. Ltd., Beijing, China) to digest cells when the cells had grown to a confluence of 70–80% in the flask (431464U, Corning, USA). The media were changed every two days, and cells at passage 3–5 were used for the following experiments. The BMSC phenotypes are identified (S Figure 2).

### 2.3. Cell Encapsulation in Matrigel

The BMSCs were encapsulated into the Matrigel according to a published method [20]. Briefly, Matrigel was diluted to 8 mg/ml with ice-cold phosphate-buffered saline (PBS), pH 7.4. The prechilled culture plates (96-well and 6-well plates) were coated with a thin layer of phenol red-free Matrigel and incubated for 20 mins at 37°C to form a layer of gel on the surface of the plates. The BMSCs were trypsinized to a single cell suspension and pelleted by centrifugation at 800 rpm for 5 mins (Sorvall, ST 16R, Thermo Fisher Scientific, USA). The BMSCs were suspended in the Matrigel at 1 × 10^6^ cells/ml. The mixture of cells and Matrigel was added to the precoated plates and further incubated at 37°C for 30 mins to allow the Matrigel to form a gel. Finally, an appropriate volume of serum-free culture media was added to the plates, and the medium was changed every two days.

### 2.4. Cell Morphology

The BMSCs incubated in the 2D plates and 3D Matrigel for 48 h were observed under an optical microscope (TE2000-U, Eclipse, Nikon) and then fixed in 4% paraformaldehyde (Aspen, China). After rinsing 3 times with PBS, the cells were stained with TRITC phalloidin (40734ES75, Yeasen, China) for 30 mins at room temperature in the dark. Then, the nuclei were counterstained with diamidine-phenylindole-dihydrochloride (DAPI) (D1306, Thermo Fisher Scientific, USA) for 5 mins, and fluorescent images were captured with a confocal fluorescence microscope system (Leica SP2, Leica, Germany).

### 2.5. Cell Viability and Proliferation

A LIVE/DEAD assay was performed to evaluate cell viability in the Matrigel and 2D plates. The LIVE/DEAD Viability/Cytotoxicity Assay kit (L3224, Invitrogen™, USA) provides a two-color fluorescence cell viability assay with calcein AM and ethidium homodimer (EthD-1). After culturing in the 2D plates or 3D Matrigel for 5 days, the BMSCs were incubated with 10 *μ*M calcein AM and 10 *μ*M EthD-1 solution at 37°C for 40 mins in the dark. The samples were then imaged under fluorescence microscopy (80i, Eclipse, Nikon). Live cells were stained green, while the dead cells were stained red. The stained cell areas were calculated with 6 different regions of interest (ROI) fields for each sample (*n* = 3) from immunofluorescence imaging using ImageJ 1.50i software (National Institutes of Health, USA).

The proliferation rates of cells cultured in Matrigel were detected by CCK-8 kits (Dojindo, Tokyo, Japan) after BMSCs were encapsulated in the Matrigel for 1, 3, 5, or 7 days. BMSCs cultured on 2D plates with normal media served as the control.

### 2.6. Osteodifferentiation Studies

BMSCs were encapsulated in the Matrigel and cultured with osteogenic media for 7 or 14 days (cell density 1 × 10^6^ cells/ml). Similar to 2D plate culture, BMSCs were seeded into the 6-well plates (same cell density as described above) with either normal media or osteogenic media (HUXMA-90021, Cyagen, USA), which contains 1 × 10^−6^ M dexamethasone, 50 *μ*g/ml ascorbic acid, and 1 × 10^−12^ M *β*-glycerophosphate. The osteogenic differentiation of BMSCs in Matrigel was assessed by ALP activity assay and Alizarin Red S staining. Briefly, 3 volumes of ice-cold PBS-EDTA was added to the Matrigel and vibrated on ice gently for 30 min until the Matrigel dissolved completely. Then, the cells were pelleted at 100×g for 5 min followed by lysing with 0.2% Triton X-100. The alkaline phosphatase (ALP) activity assay was performed following the manufacturer's instructions (Beyotime, China) in the presence of the substrate p-nitrophenyl phosphate (pNPP). The final ALP activity was normalized as pNPP production (nM) per total intracellular protein content (mg) per min (nmol/min/mg protein) (determined by MicroBCA protein assay kits, Thermo Fisher Scientific).

After incubation for 14 days, all cells were stained with a 0.1% Alizarin Red S (Sigma-Aldrich, USA) for calcium deposits, and an optical microscope (TE2000-U, Eclipse, Nikon) was used to take photographs. To quantify the orange-red coloration of ARS, 10% acetic acid (Sigma Aldrich, USA) was added to the plates. After centrifuging and neutralizing with 10% ammonium hydroxide (Sigma-Aldrich, USA), 100 *μ*l of each sample was added to 96-well plates, and the OD405 was read using a microplate reader (Varioskan Flash, Thermo Fisher Scientific).

### 2.7. Western Blotting Analyses

BMSCs were encapsulated and cultured with osteogenic media in Matrigel or cultured in the 6-well plates with either normal media or osteogenic media as described above. Total proteins were isolated from these cells after incubation for 14 days. An equal amount of protein (50 *μ*g protein/lane) was separated by sodium dodecyl sulfate polyacrylamide gel electrophoresis (SDS-PAGE) and transferred to polyvinylidene difluoride (PVDF) membranes. The membranes were incubated in 5% skimmed milk for 1 hour at room temperature and overnight at 4°C with primary rabbit antibodies. All antibodies were purchased from Abcam Inc. (UK). GAPDH was used as the control. Bands were visualized using an ECL chemiluminescent kit (WP20005, Invitrogen™, USA) and quantitated by Quantity One (Bio-Rad, Hercules, USA).

### 2.8. Real-Time Quantitative PCR

RT-PCR was used to assess the osteogenic mRNA expression of BMSCs. At day 7 and day 14, RNA was extracted from encapsulated BMSCs cultured with osteogenic media in Matrigel or 6-well plates (cultured with either normal media or osteogenic media). Briefly, total RNA was extracted with TRIZOL reagent (Invitrogen™, USA) and reverse transcribed into cDNA using a cDNA synthesis kit (Thermo Fisher Scientific, USA) according to the manufacturer's protocol. PCR conditions comprised an initial step of denaturation for 1 min at 95°C, followed by a total of 40 cycles of 15 s at 95°C, 20 s at 58°C, and 20 s at 72°C. The expression levels of osteoblastic markers, including Col-1, Runx2, OPN, ALP, and OCN, were calculated based on the 2^−∆∆Ct^ method by normalizing values to the housekeeping gene GAPDH. The primer sequences of selected genes are listed in Table 1.

### 2.9. Surgical Procedures

Forty-eight male SD rats weighing 250–300 g were purchased from the Laboratory Animal Center of Peking Union Medical College Hospital (PUMCH) and were used for animal experiments. All experimental procedures were approved by the Institutional Animal Care and Use Committee (IACUC) of PUMCH. All the rats were anesthetized with ketamine hydrochloride (6 mg/100 g) and a 5 mm full-thickness standardized defect was made with a stainless-steel punch on the mandibular ramus of SD rats (S Figure 1A and B). All rats were randomly divided into four groups (*n* = 12 each group): (1) bare scaffold (BS); (2) scaffold with injected BMSCs + PBS (SC); (3) scaffold + Matrigel (SM); and (4) scaffold + BMSC-loaded Matrigel (SMB). For the BS group, the bare scaffold was carefully pressed to fit into the defect. For the SC group, 1 × 10^6^ BMSCs were resuspended in 250 *μ*l PBS and then injected into the scaffold after implantation. For the SMB group and the SM group, Matrigel with or without 1 × 10^6^ encapsulated BMSCs was added to fill the scaffold before implantation (S Figure 1C and D). Finally, the wounds were closed with sutures. Soft gel food was provided, and penicillin was intramuscularly injected for three days postsurgery.

### 2.10. Micro-CT Evaluation

At 6 and 12 weeks postoperatively, the mandibles were harvested. The mandibles were first fixed in 4% paraformaldehyde and then dehydrated in a graded ethanol solution from 70%–100% and embedded in methyl methacrylate. Micro-CT scans were performed using inspeXio SMX-90CT Plus micro-CT (SHIMADZU, Japan) with a resolution of 10 *μ*m (voltage: 90 kV, current: 110 *μ*A, 1.0 mm Al filter). The volume of newly formed hard tissue within the defect area was assessed through MIMICS 15.0 (Materialise, Belgium), and bone regeneration was expressed as bone volume (BV), total volume (TV), and percent bone volume (BV/TV).

### 2.11. Histological Analysis

An interlocked diamond saw (Leica Microtome, Wetzlar, Germany) was used to obtain 200 *μ*m thick sections. After polishing to a thickness of 50 *μ*m, these sections were stained in 1.2% trinitrophenol solution as well as 1% acid fuchsin solution (Van Gieson staining). The stained sections were observed under an optical microscope (TE2000-U, Eclipse, Nikon). The red represents new bone formation, the blue indicates fibrous tissue, and the black is the metal.

### 2.12. Statistical Analysis

All the values were expressed as the mean ± standard deviation (SD). Statistical significance was measured using Student's *t*-test and one-way analysis of variance (ANOVA). Statistical significance was defined as *P* value < 0.05.

## 3. Results

### 3.1. Cell Culture and Morphology

The cells in the 2D plate showed a spindle-like morphology and were well spread in the wells. Compared with 2D plate culture, the BMSCs in the Matrigel exhibited more connections with surrounding cells, and separated cell clusters can be seen (Figures 2(a) and 2(b)). The cell adhesion and connection behavior were investigated by fluorescent staining of F-actin (by rhodamine-phalloidin, shown as red) and nuclei (by DAPI, shown as blue), respectively. The BMSCs within the Matrigel formed a more complex network and interactions between them than did the 2D plate culture (Figures 2(c) and 2(d)). For quantitative analysis of cell adhesion and interaction behavior, the cell nuclei number and fluorescent intensity were measured using ImageJ software. The results showed that both the fluorescent intensity of F-actin and the cell nuclei number in the 3D Matrigel group were significantly higher than in the 2D plated culture (Figures 2(e) and 2(f)) (fluorescent intensity of F-actin *P* < 0.01 and nuclei number *P* < 0.05). This result indicated that the 3D Matrigel culture environment could promote BMSC adherence and interaction with surrounding cells.

### 3.2. Cell Proliferation and Biocompatibility

**Figure 3:**
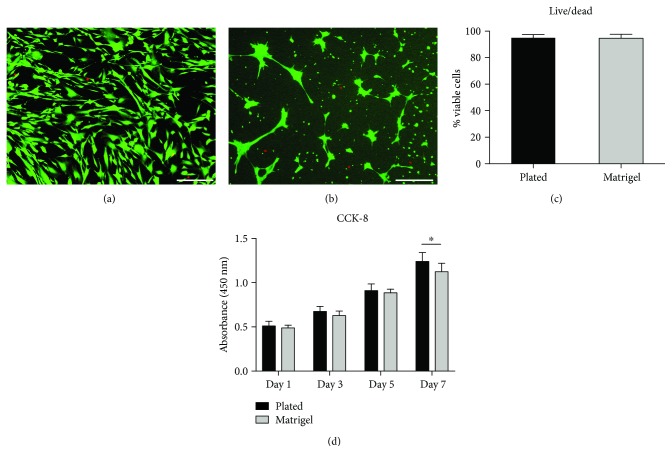
Representative images of the live/dead assay of BMSCs cultured on 2D plates (a) and encapsulated within the Matrigel (b) at 5 days (scale bar: 150 *μ*m). The quantitative results of the live/dead assay of BMSCs (c). A CCK8 assay was used to assess BMSC proliferation over 7 days (d). ^∗^ *p* < 0.05.

Figures 3(a) and 3(b) show the LIVE/DEAD staining images after culturing for 5 days in Matrigel or 2D plates. Almost no dead cells can be seen in either group. The fluorescence intensity of the cells calculated by ImageJ software showed that no differences were found between the two groups (Figure 3(c)). BMSC proliferation was investigated by CCK-8 assay at day 1, 3, 5, and 7 after incubation. The cell viability steadily increased both in 2D plate culture and 3D Matrigel encapsulated culture during the incubation period (Figure 3(d)). These results indicate that the Matrigel can support BMSC proliferation and showed a negligible reduction in cell viability.

### 3.3. ALP Activity and ARS Staining

**Figure 4:**
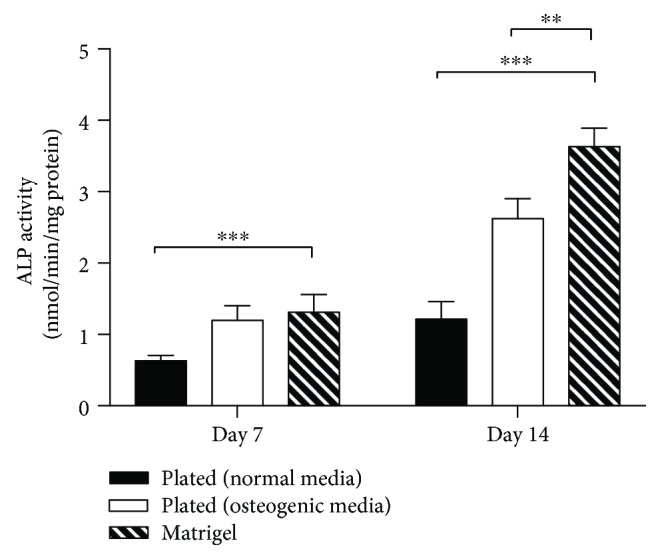
ALP activity of BMSCs cultured on 2D plates with standard medium or with osteogenic medium and BMSCs encapsulated in 3D Matrigel after culture for 7 and 14 days. ^∗∗^ *p* < 0.01, ^∗∗∗^ *p* < 0.001.

Alkaline phosphatase plays an important role in the matrix mineralization process. We evaluated the bone differentiation capability of BMSCs by ALP activity after 7 and 14 days of incubation. With increasing incubation time, the ALP activity of the experimental groups gradually increased. It should be noted that there was no significant difference between 2D culture induced by osteogenic media and 3D Matrigel culture at day 7. However, at day 14, the ALP activity in the Matrigel group was significantly higher than that in the other two groups (*P* < 0.001) (Figure 4).

**Figure 5:**
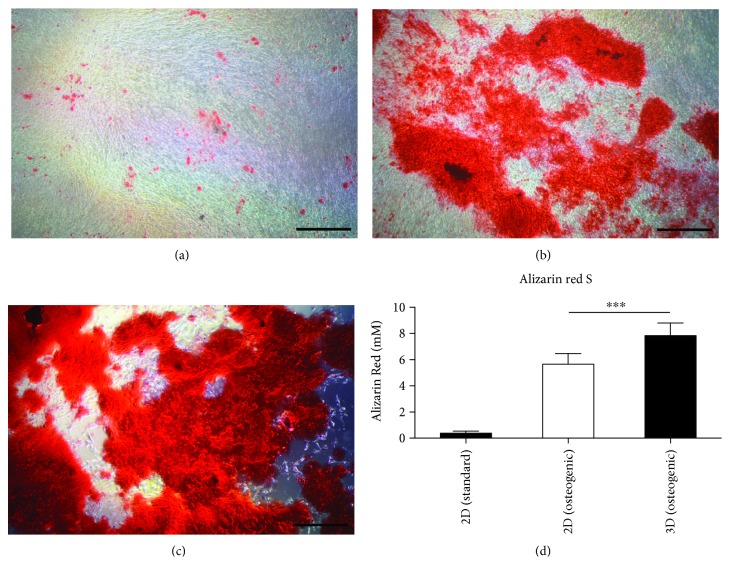
The amount of the mineralized matrix deposited was determined by Alizarin Red S staining and quantification on day 14. BMSC 2D culture with standard medium (a), BMSC 2D culture with osteogenic medium (b), BMSCs cultured in 3D Matrigel (c), and quantitative levels of calcium accumulation (d). ^∗∗∗^ *p* < 0.001.

Alizarin Red S was used to identify the calcium deposition of the BMSCs after incubation in 2D plates and 3D Matrigel. Figures 5(a)–5(c) show the deposition of the mineralized matrix on day 14. After extraction with acetic acid, a quantitative analysis of the formed calcium nodes was undertaken by colorimetric detection (Figure 5(d)). The results showed that the osteogenic medium significantly improved the cellular mineralization ability, and when compared to the 2D plates with an osteogenic medium, cells in the 3D Matrigel exhibited nearly 1.5 times higher calcium node deposition. These results indicated that 3D Matrigel culture could promote osteogenic differentiation of BMSCs.

### 3.4. Induction of BMSC Differentiation by Matrigel

**Figure 6:**
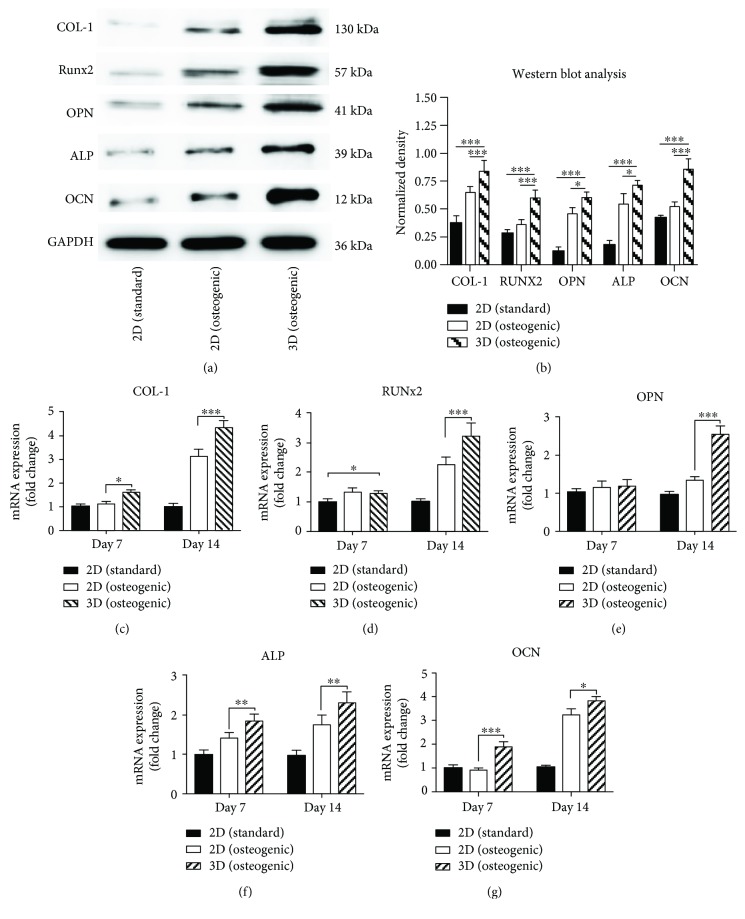
Western blot analysis was used to determine the expression of COL-I, Runx2, OPN, ALP, and OCN in BMSCs (a). Quantification of the density of Western blots in each group (b). The real-time polymerase chain reaction (RT-PCR) measurements of the COL-1 (c), Runx2 (d), OPN (e), ALP (f), and OCN (g) genes. These values are ratios relative to GAPDH and the mean relative quantity (RQ) ± square deviation (SD). ^∗^ *p* < 0.05, ^∗∗^ *p* < 0.01, ^∗∗∗^ *p* < 0.001.

Western blot assays were used to evaluate osteogenesis markers, including Col-1, Runx2, OPN, ALP, and OCN, while GAPDH was used as a housekeeping gene. After 14 days of culture in Matrigel with the osteogenic medium, the expression level of all osteogenic-related proteins, such as Col-1, Runx2, and OCN (*P* < 0.001) and OPN and ALP (*P* < 0.05), in BMSCs was much higher when compared to the cells cultured in 2D plates with the osteogenic medium. All these proteins were significantly higher in 3D Matrigel-cultured BMSCs compared to those cultured in 2D standard medium (*P* < 0.001) (Figures 6(a) and 6(b)).

The RT-PCR test was performed on day 7 and day 14 to evaluate the mRNA expression levels of Col-1, Runx2, OPN, ALP, and OCN. On day 7, 3D-cultured BMSCs showed higher mRNA expression of Col-1 (*P* < 0.05), ALP (*P* < 0.01), and OCN (*P* < 0.001) than 2D-cultured BMSCs with the osteogenic medium. Similarly, RT-PCR analysis confirmed the same results as those observed by Western blotting on day 14, which showed significantly upregulated expression of Col-1 (*P* < 0.001), Runx2 (*P* < 0.001), OPN (*P* < 0.001), ALP (*P* < 0.01), and OCN (*P* < 0.05) in the 3D Matrigel group (Figures 6(c)–6(g)). These results confirmed the osteogenic differentiation ability of Matrigel at both the protein and mRNA levels.

### 3.5. Micro-CT Analysis

**Figure 7:**
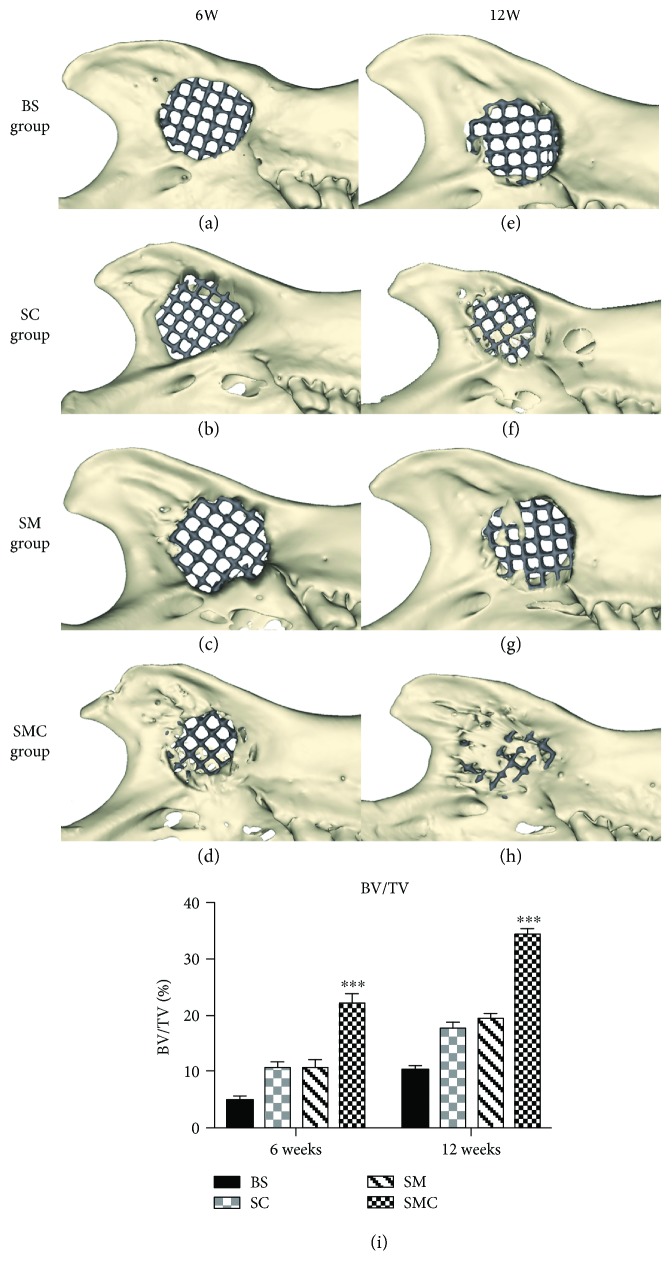
The volume of regenerated bone reconstruction based on ex vivo micro-CT scans at 6 and 12 weeks (a–h). The percent bone volume BV/TV was calculated by MIMICS 15.0 (Materialise, Belgium) (i). ^∗∗∗^ *p* < 0.001.

No complications were observed in the 48 SD rats, and all skin wounds were healed within 5 days. At 6 and 12 weeks after implantation, micro-CT was used to detect the newly formed bone within the scaffold. As shown in Figure 7, the BS group without Matrigel and stem cells showed rare bone formation. The Matrigel alone and the BMSC injection resulted in only minor neobone formation. However, the SMC group exhibited accelerated bone regeneration and showed clear enhancement of the repair as determined by a quantitative analysis of BV/TV (Figure 7(i)).

### 3.6. Histological Evaluation

**Figure 8:**
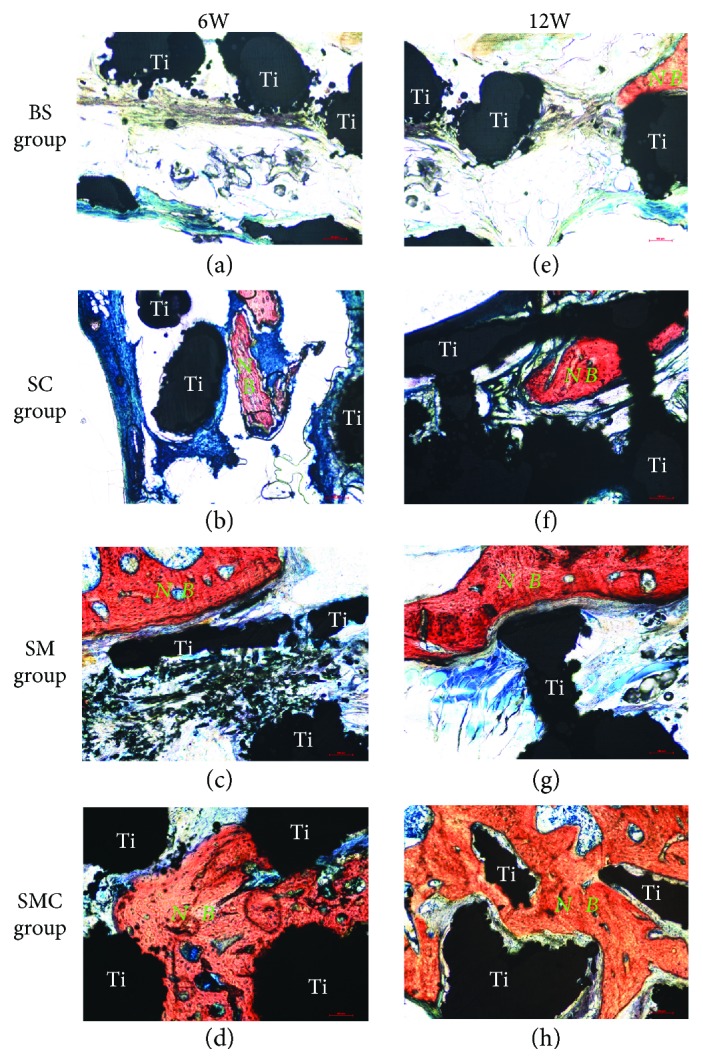
Histological analysis of osteointegration ability of four groups using Van Gieson staining at 6 weeks (a, b, c, and d) and 12 weeks (e, f, g, and h). BS group (a, e); SC group (b, f); SM group (c, g); SMC group (d, h). (red represents new bone formation (NB), blue indicates fibrous tissue, and black is the metal (Ti)) (scale bar: 100 *μ*m).

Histological analyses were used to characterize mineralized formation in all defects. At the end of 6 and 12 weeks, the rats were euthanized, and the implant site tissue was harvested for Van Gieson staining. The histological analysis results corresponded with those observed by micro-CT. The amount of the new bone was significantly higher in the SMC group compared with that in the other three groups both at 6 and 12 weeks, and the pores of the Ti6Al4V scaffold were filled with the newly formed bone. The newly formed bone was found rarely in the BS group. In the SM group, the new bone was only found at the surface of the scaffold and not inside. In the SC group, only a few newly formed bones were detected within the scaffold (Figure 8).

## 4. Discussion

Over the past decade, great progress has been made in the surgical techniques and clinical outcomes of mandibular defect repair. However, mandibular bone regeneration remains a major unsolved problem. The lack of osteogenic cells in the surgical bed continues to be a pitfall in every mandibular reconstruction [21]. In this study, Matrigel was chosen to provide an excellent BMSC 3D culture environment for bone regeneration, and the combination of 3D-printed Ti6Al4V scaffold can serve as a complementary component to provide robust bone structure and customized shape.

### 4.1. Matrigel Increases BMSC Osteogenic Activity *In Vitro*

Critical mandibular defects need a considerable number of BMSCs. However, BMSCs are scarce in tissue [22]. Thus, BMSC grafts are considered to be a promising method for treating mandibular bone defects. Keeping BMSCs viable and promoting bone differentiation is a major concern. In the present study, BMSCs were encapsulated in Matrigel for 3D culture, and BMSCs grown on 2D plates either in normal or osteogenic media were used as controls. Interestingly, the BMSCs grown in Matrigel showed a higher level of ALP activity, calcium deposition, and osteogenic-related protein and gene expression than those cultured on 2D plates with or without osteogenic media. A previous study showed that stem cells grown on Matrigel-coated surfaces had higher osteogenic activity [19]. This is because of the ECM properties of Matrigel, which can promote the osteogenic differentiation of BMSCs directly. The ECM has already been reported to provide structural support for cells and physical and biochemical cues to regulate cell phenotype [23, 24]. BMSCs cultured in Matrigel can interact with multiple ECM components within the Matrigel, such as laminin and TGF-*β*. The laminin can regulate osteogenic differentiation via the integrin signaling pathway [25], and TGF-*β* is an important osteogenic growth factor.

Moreover, 3D culture in Matrigel strongly mimics the physiological condition *in vivo*. Many studies have reported that 3D culture provides an optimal environment for cellular adhesion and communication [23, 26]. The 3D constructs represent a completely different environment, due to different signaling pathway activity compared to 2D culture. Grayson et al. [27] reported that BMSCs express more integrin in 3D culture compared to that in 2D culture. Thus, 3D culture might activate the integrin-BMP/Smad pathway in BMSCs [28]. However, traditional 2D-cultured stem cells are grown on plastic surfaces, which helps them to retain proliferation potential but dramatically reduces differentiation capacity [23]. Previous reports have proposed that culturing BMSCs in a 3D hydrogel system could reduce their stemness properties and differentiate them into other lineages, such as osteogenic or myogenic commitment [9, 29]. This agrees with the results of the CCK-8 assay, where the BMSCs cultured in the Matrigel proliferated less than those on the 2D plates (Figure 3(d)). Therefore, some researchers have reported that the differentiation capability of stem cells is enhanced in a 3D culture system both *in vivo* and *in vitro* [30, 31]. In the present study, we also found that the 3D Matrigel culture could promote cell adhesion and that the Matrigel served as an artificial 3D microenvironment to regulate BMSC bone differentiation *in vitro*. However, the underlying mechanisms require further exploration.

### 4.2. Scaffold-Matrigel-BMSC Complex Enhances Bone Repair *In Vivo*

The reconstruction of the mandible is different from midface or calvaria repair because it demands load-bearing ability. Ti has been attractive for clinical use because of its excellent load-bearing properties, biocompatibility, and high corrosion resistance [32]. Today, with the help of digital medicine and computer-aided technology, a 3D-printed porous Ti scaffold could mimic bone tissue morphology and mechanical behavior functionally and aesthetically, thus helping to reconstruct mandibular bone defects [33, 34]. The 3D-printed implants can reconstruct complex defective sections anatomically using a mirror image of the unaffected side [34, 35]. In this study, 3D-printed Ti6Al4V scaffolds with more than 80% porosity can accommodate new bone and provide an adequate biological fixation. However, three major factors were found to restrict bone regeneration, including the lack of stem cells, the bioinert properties of Ti, and the intrusion of the surrounding muscle into the scaffold. To solve these problems, BMSC-loaded Matrigels were prepared and incorporated into the 3D-printed porous Ti6Al4V scaffold. Therefore, we hypothesized that by combining cell therapy and 3D-printed scaffolds, the device might provide a robust bone substitute with biological activity for bone regeneration.

To evaluate the hypothesis, a mandibular ramus defect model of 5 mm was established to fully investigate the bone repair of BMSC-loaded Matrigel + porous Ti6Al4V scaffold *in vivo*. The micro-CT and histological analysis results both confirmed the bone repair capability of our device. In the animal model, a defect diameter greater than 5 mm is a critical size that cannot spontaneously heal [36–38]. The graft BMSCs could help bone formation within the scaffold. After 12 weeks of device implantation, both micro-CT and histological analysis showed that the bare scaffold group has rare bone formation (Figures 7(e) and 8(e)). However, in the scaffold + injected BMSCs group, few new bones formed within the scaffold (Figures 7(f) and 8(f)). This indicated that the BMSC-injection grafting method could partially help new bone formation, but the low survival rate of grafted BMSCs, which results from acute inflammation and mechanical damage, is the major obstacle [11]. Therefore, we encapsulated BMSC in Matrigel because Matrigel can retain BMSC viability, enhance the osteogenic potential of BMSCs, and prevent surrounding muscle intrusion into the scaffold. Cao et al. [39] used longitudinal bioluminescence imaging to assess cell signals in the Matrigel and found that Matrigel supported stem cell engraftment superior to cells alone or other matrices (collagen I and Puramatrix) up to 5 months. Thus, we believe that BMSCs may promote bone repair up to 5 months. Rosová et al. [40] and Chen et al. [41] reported that hypoxic conditions could improve BMSC survival and tissue regenerative potential *in vivo*, and the Matrigel hydrogel microenvironment is moderately hypoxic. Similarly, in our scaffold + Matrigel group, Matrigel without BMSCs inside only formed new bone on the surface of the scaffold, not inside (Figures 7(g) and 8(g)). However, in the scaffold + BMSC-loaded Matrigel group, BMSC viability showed a dramatic increase, and a large amount of new bone tissue formed around and within the scaffold (Figures 7(h) and 8(h)). This method not only solved the abovementioned disadvantages of the Ti scaffold but also produced a scaffold-Matrigel-BMSC complex with appropriate bioactivity and mechanical properties that enhanced bone repair *in vivo*. However, the long-term function of BMSCs in the *in vivo* model need further investigation before clinical usage.

In conclusion, this combination device was shown to be a complementary treatment modality for both Ti scaffold- and hydrogel-based cell therapy by avoiding the bioinertia of Ti scaffold and the weak mechanical properties of Matrigel. We believe that this device might be a promising method with high stem cell therapy efficiency for the treatment of mandibular bone defects. For further applications, a clinical trial is needed in the near future.

## Figures and Tables

**Table 1:** Primer pair sequences for the studied genes.

Gene	Sequences		Annealing temperature (°C)
	Forward (5′-3′)	Reverse (3′-5′)	
GAPDH	GTCAAGGCTGAGAACGGGAA	AAATGAGCCCCAGCCTTCTC	58
COL-1	CCTCGCTTTCCTTCCTCTCC	GTCAAGGCTGAGAACGGGAA	58
Runx2	CATGTCCCTCGGTATGTCCG	ACTCTGGCTTTGGGAAGAGC	58
OPN	ATCTCCTAGCCCCACAGACCC	CACACTATCACCTCGGCCATC	58
ALP	TCCTGTTGACACCCCAAACC	GGAAACGCAGGATTTCCCAC	58
OCN	CTCACACTCCTCGCCCTATTG	CGCCTGGGTCTCTTCACTAC	58

## Data Availability

The data used to support the findings of this study are available from the corresponding author upon request.

## Supplementary Materials

Supplementary Materials Figure 1: the surgical procedure for the rat mandibular defect model. Exposure of the mandibular ramus (A). 5 mm full-thickness standardized defect was made (B). Porous titanium scaffold was pressed into the defect (C). Gross appearance of the mandibular defect (D). Supplementary Figure 2: BMSC phenotypes were identified. CD34− cells accounted for 0.05% of total cells. CD44+ cells accounted for 98.65% of total cells. CD45− cells accounted for 0.23% of total cells. CD73+ cells accounted for 99.73% of total cells. CD90+ cells accounted for 95.69% of total cells. CD105+ cells accounted for 97.32% of total cells. HLA-ABC+ cells accounted for 99.87% of total cells. HLA-DR− cells accounted for 0.04% of total cells.

## References

[1] Larsson L., Decker A. M., Nibali L., Pilipchuk S. P., Berglundh T., Giannobile W. V. (2016). Regenerative medicine for periodontal and peri-implant diseases. *Journal of Dental Research*.

[2] Abbasi A. J., Azari A., Mohebbi S. Z., Javani A. (2017). Mandibular rami implant: a new approach in mandibular reconstruction. *Journal of Oral and Maxillofacial Surgery*.

[3] Goh B. T., Lee S., Tideman H., Stoelinga P. J. W. (2008). Mandibular reconstruction in adults: a review. *International Journal of Oral and Maxillofacial Surgery*.

[4] Wang X., Wu X., Xing H. (2017). Porous Nanohydroxyapatite/collagen scaffolds loading insulin PLGA particles for restoration of critical size bone defect. *ACS Applied Materials & Interfaces*.

[5] Yin B., Ma P., Chen J. (2016). Hybrid macro-porous titanium ornamented by degradable 3D gel/nHA micro-scaffolds for bone tissue regeneration. *International Journal of Molecular Sciences*.

[6] Shao H., Sun M., Zhang F. (2018). Custom repair of mandibular bone defects with 3D printed bioceramic scaffolds. *Journal of Dental Research*.

[7] Van der Stok J., Van Lieshout E. M. M., El-Massoudi Y., Van Kralingen G. H., Patka P. (2011). Bone substitutes in the Netherlands – a systematic literature review. *Acta Biomaterialia*.

[8] Raisian S., Fallahi H. R., Khiabani K. S., Heidarizadeh M., Azdoo S. (2017). Customized titanium mesh based on the 3D printed model vs. manual intraoperative bending of titanium mesh for reconstructing of orbital bone fracture: a randomized clinical trial. *Reviews on Recent Clinical Trials*.

[9] Shou K., Huang Y., Qi B. (2018). Induction of mesenchymal stem cell differentiation in the absence of soluble inducer for cutaneous wound regeneration by a chitin nanofiber-based hydrogel. *Journal of Tissue Engineering and Regenerative Medicine*.

[10] Duan Y., Ma W., Li D., Wang T., Liu B. (2017). Enhanced osseointegration of titanium implants in a rat model of osteoporosis using multilayer bone mesenchymal stem cell sheets. *Experimental and Therapeutic Medicine*.

[11] Uemura M., Refaat M. M., Shinoyama M., Hayashi H., Hashimoto N., Takahashi J. (2010). Matrigel supports survival and neuronal differentiation of grafted embryonic stem cell-derived neural precursor cells. *Journal of Neuroscience Research*.

[12] Führmann T., Tam R. Y., Ballarin B. (2016). Injectable hydrogel promotes early survival of induced pluripotent stem cell-derived oligodendrocytes and attenuates longterm teratoma formation in a spinal cord injury model. *Biomaterials*.

[13] Braccini A., Wendt D., Jaquiery C. (2005). Three-dimensional perfusion culture of human bone marrow cells and generation of osteoinductive grafts. *Stem Cells*.

[14] Kim S. E., Shim K. M., Jang K., Shim J. H., Kang S. S. (2018). Three-dimensional printing-based reconstruction of a maxillary bone defect in a dog following tumor removal. *In Vivo*.

[15] Qian L., Saltzman W. M. (2004). Improving the expansion and neuronal differentiation of mesenchymal stem cells through culture surface modification. *Biomaterials*.

[16] Xiao Q., Zeng L., Zhang Z. (2006). Sca-1^+^ progenitors derived from embryonic stem cells differentiate into endothelial cells capable of vascular repair after arterial injury. *Arteriosclerosis, Thrombosis, and Vascular Biology*.

[17] Zhang P., Zhang H., Wang H., Wei Y., Hu S. (2006). Artificial matrix helps neonatal cardiomyocytes restore injured myocardium in rats. *Artificial Organs*.

[18] Kang B.-J., Ryu H.-H., Park S.-S. (2012). Effect of matrigel on the osteogenic potential of canine adipose tissue-derived mesenchymal stem cells. *Journal of Veterinary Medical Science*.

[19] Eslaminejad M. B., Bagheri F., Zomorodian E. (2010). Matrigel enhances *in vitro* bone differentiation of human marrow-derived mesenchymal stem cells. *Iranian Journal of Basic Medical Sciences*.

[20] Lee G. Y., Kenny P. A., Lee E. H., Bissell M. J. (2007). Three-dimensional culture models of normal and malignant breast epithelial cells. *Nature Methods*.

[21] Fu K., Liu Y., Gao N., Cai J., He W., Qiu W. (2017). Reconstruction of maxillary and orbital floor defect with free fibula flap and whole individualized titanium mesh assisted by computer techniques. *Journal Oral and Maxillofacial Surgery*.

[22] Pittenger M. F., Mackay A. M., Beck S. C. (1999). Multilineage potential of adult human mesenchymal stem cells. *Science*.

[23] Zhang Q., Lin S., Liao J., Cai X. (2018). Physical cues drive chondrogenic differentiation. *Current Stem Cell Research & Therapy*.

[24] Cheng A., Cain S. A., Tian P. (2018). Recombinant extracellular matrix protein fragments support human embryonic stem cell chondrogenesis. *Tissue Engineering Part A*.

[25] Viale-Bouroncle S., Gosau M., Morsczeck C. (2014). Laminin regulates the osteogenic differentiation of dental follicle cells via integrin-α2/-β1 and the activation of the FAK/ERK signaling pathway. *Cell and Tissue Research*.

[26] Gupta D., Grant D. M., Zakir Hossain K. M., Ahmed I., Sottile V. (2018). Role of geometrical cues in bone marrow-derived mesenchymal stem cell survival, growth and osteogenic differentiation. *Journal of Biomaterials Applications*.

[27] Grayson W. L., Ma T., Bunnell B. (2004). Human mesenchymal stem cells tissue development in 3D PET matrices. *Biotechnology Progress*.

[28] Liu H., Peng H., Wu Y. (2013). The promotion of bone regeneration by nanofibrous hydroxyapatite/chitosan scaffolds by effects on integrin-BMP/Smad signaling pathway in BMSCs. *Biomaterials*.

[29] Tseng P. C., Young T. H., Wang T. M., Peng H. W., Hou S. M., Yen M. L. (2012). Spontaneous osteogenesis of MSCs cultured on 3D microcarriers through alteration of cytoskeletal tension. *Biomaterials*.

[30] Tian X.‐. F., Heng B. C., Ge Z. (2009). Comparison of osteogenesis of human embryonic stem cells within 2D and 3D culture systems. *Scandinavian Journal of Clinical and Laboratory Investigation*.

[31] Rath S. N., Nooeaid P., Arkudas A. (2016). Adipose- and bone marrow-derived mesenchymal stem cells display different osteogenic differentiation patterns in 3D bioactive glass-based scaffolds. *Journal of Tissue Engineering and Regenerative Medicine*.

[32] Suska F., Kjeller G., Tarnow P. (2016). Electron beam melting manufacturing technology for individually manufactured jaw prosthesis: a case report. *Journal of Oral and Maxillofacial Surgery*.

[33] Schouman T., Schmitt M., Adam C., Dubois G., Rouch P. (2016). Influence of the overall stiffness of a load-bearing porous titanium implant on bone ingrowth in critical-size mandibular bone defects in sheep. *Journal of the Mechanical Behavior of Biomedical Materials*.

[34] Luo D., Rong Q., Chen Q. (2017). Finite-element design and optimization of a three-dimensional tetrahedral porous titanium scaffold for the reconstruction of mandibular defects. *Medical Engineering & Physics*.

[35] Leiser Y., Shilo D., Wolff A., Rachmiel A. (2016). Functional reconstruction in mandibular avulsion injuries. *The Journal of Craniofacial Surgery*.

[36] Zhang W., Zhang Z., Chen S., Macri L., Kohn J., Yelick P. C. (2016). Mandibular jaw bone regeneration using human dental cell-seeded tyrosine-derived polycarbonate scaffolds. *Tissue Engineering Part A*.

[37] Kaban L. B., Glowacki J., Murray J. E. (1979). Repair of experimental mandibular bony defects in rats. *Surgical Forum*.

[38] Wang X., Xing H., Zhang G. (2016). Restoration of a critical mandibular bone defect using human alveolar bone-derived stem cells and porous nano-HA/collagen/PLA scaffold. *Stem Cells International*.

[39] Cao F., Sadrzadeh Rafie A. H., Abilez O. J. (2007). In vivo imaging and evaluation of different biomatrices for improvement of stem cell survival. *Journal of Tissue Engineering and Regenerative Medicine*.

[40] Rosová I., Dao M., Capoccia B., Link D., Nolta J. A. (2008). Hypoxic preconditioning results in increased motility and improved therapeutic potential of human mesenchymal stem cells. *Stem Cells*.

[41] Chen J., Yang Y., Shen L. (2017). Hypoxic preconditioning augments the therapeutic efficacy of bone marrow stromal cells in a rat ischemic stroke model. *Cellular and Molecular Neurobiology*.

