# Supplementary material for: 3D Culture of Bone Marrow-Derived Mesenchymal Stem Cells (BMSCs) Could Improve Bone Regeneration in 3D-Printed Porous Ti6Al4V Scaffolds
Source: Stem Cells Int. 2018 Sep 5;2018:2074021. doi: 10.1155/2018/2074021 (PMC6145055; doi:10.1155/2018/2074021)
Supplement: Supplementary Materials — Figure 1: the surgical procedure for the rat mandibular defect model. Exposure of the mandibular ramus (A). 5 mm full-thickness standardized defect was made (B). Porous titanium scaffold was pressed into the defect (C). Gross appearance of the mandibular defect (D). Supplementary Figure 2: BMSC phenotypes were identified. CD34− cells accounted for 0.05% of total cells. CD44+ cells accounted for 98.65% of total cells. CD45− cells accounted for 0.23% of total cells. CD73+ cells accounted for 99.73% of total cells. CD90+ cells accounted for 95.69% of total cells. CD105+ cells accounted for 97.32% of total cells. HLA-ABC+ cells accounted for 99.87% of total cells. HLA-DR− cells accounted for 0.04% of total cells. [file 2074021.f1.docx]

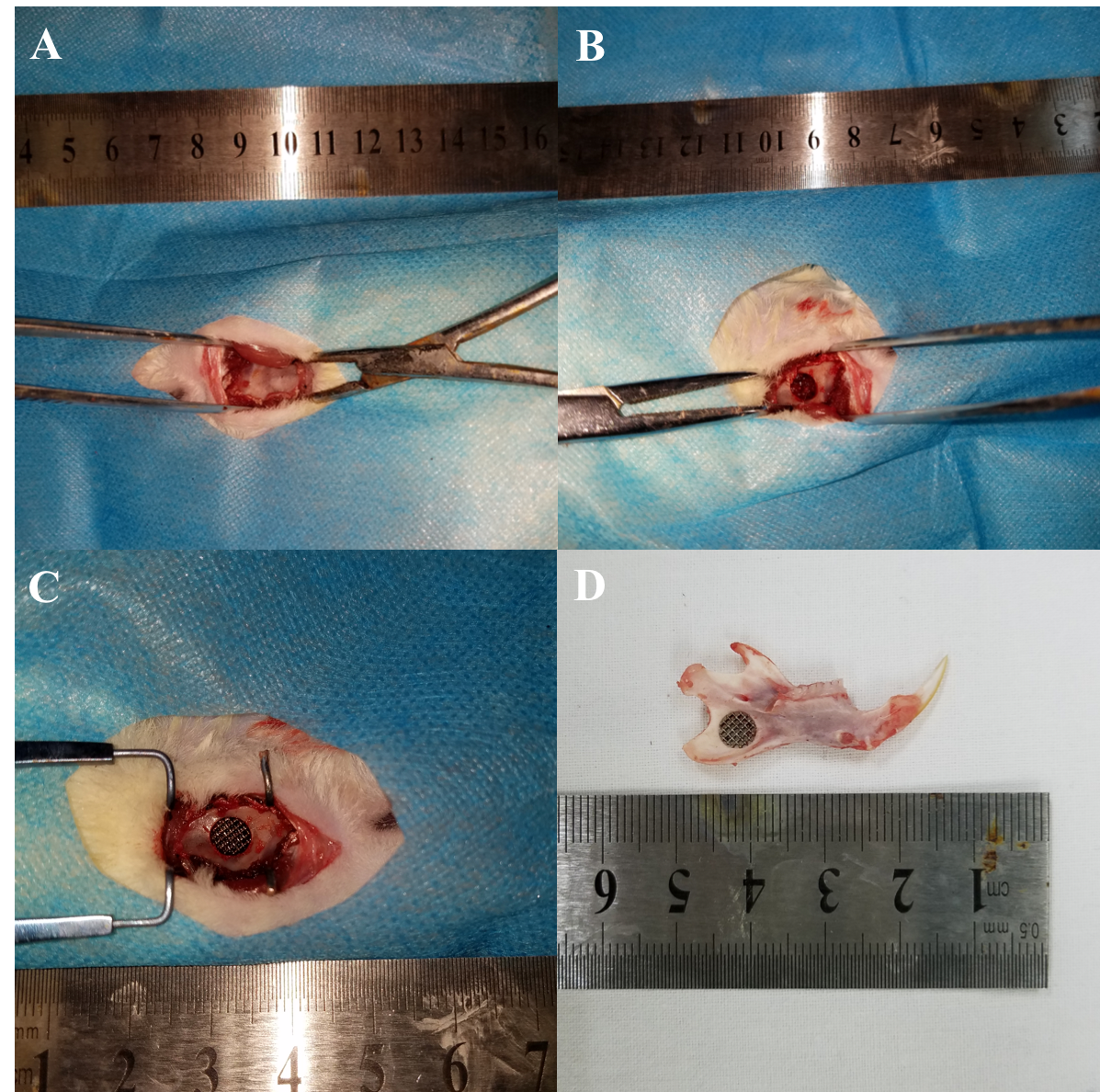


Supplementary Figure 1：The surgical procedure for the rat mandibular defect model. Exposure of the mandibular ramus(A). 5mm full-thickness standardized defect was made(B). Porous titanium scaffold was pressed into the defect(C). Gross appearance of the mandibular defect(D).


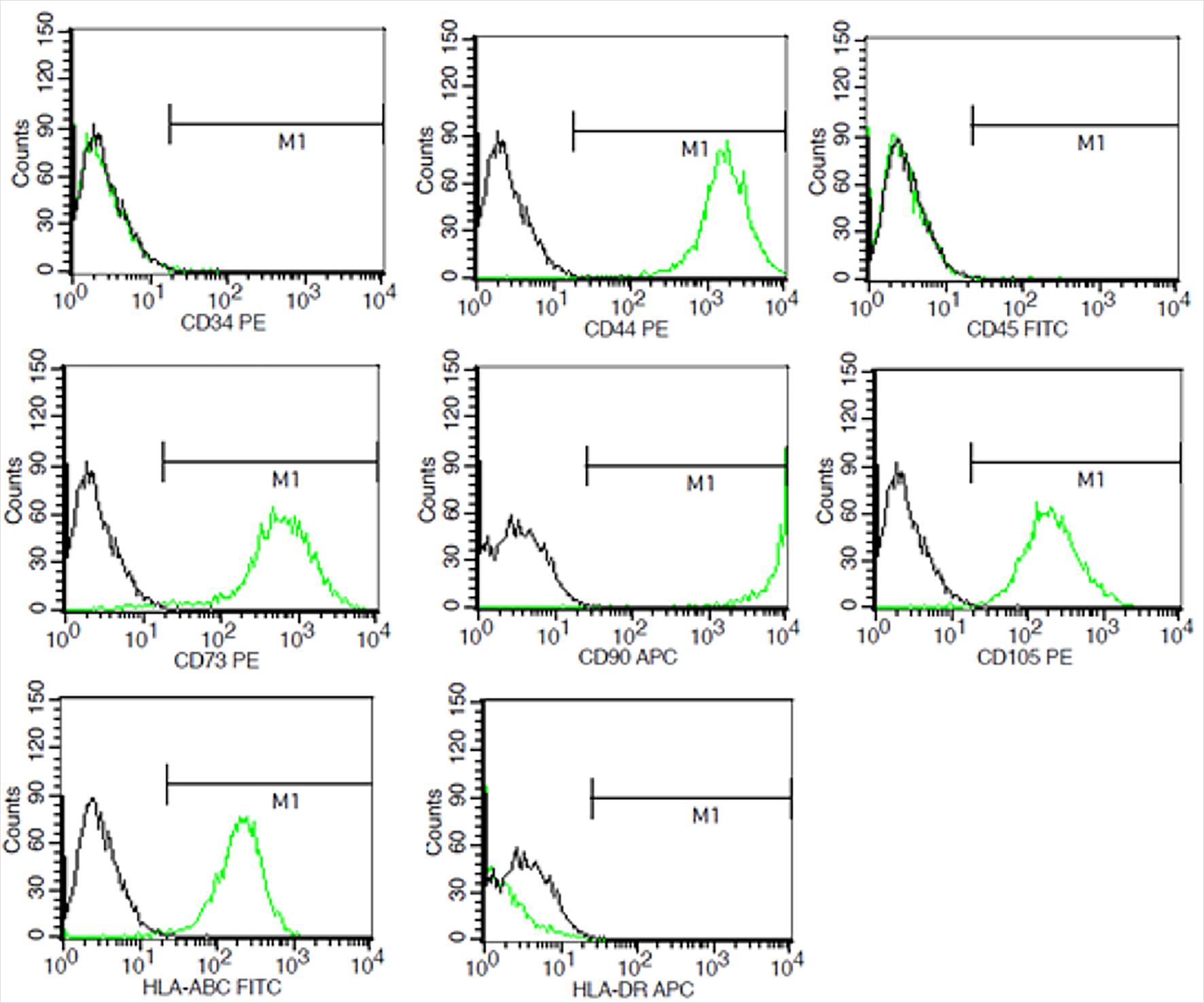


Supplementary Figure 2: BMSC phenotypes were identified. CD34- cells accounted for 0.05% of total cells. CD44+ cells accounted for 98.65% of total cells. CD45- cells accounted for 0.23% of total cells. CD73+ cells accounted for 99.73% of total cells. CD90+ cells accounted for 95.69% of total cells. CD105+ cells accounted for 97.32% of total cells. HLA-ABC+ cells accounted for 99.87% of total cells. HLA-DR- cells accounted for 0.04% of total cells.
